# The development of a GeXP-based multiplex reverse transcription-PCR assay for simultaneous detection of sixteen human respiratory virus types/subtypes

**DOI:** 10.1186/1471-2334-12-189

**Published:** 2012-08-14

**Authors:** Jin Li, Nai-Ying Mao, Chen Zhang, Meng-Jie Yang, Miao Wang, Wen-Bo Xu, Xue-Jun Ma

**Affiliations:** 1 State Key Laboratory for Molecular Virology and Genetic Engineering, National Institute for Viral Disease Control and Prevention, Chinese Center for Disease Control and Prevention, Changbai Rd 155, Beijing, Changping District, 102206, China

**Keywords:** GeXP, Human respiratory viruses, Multiplex RT-PCR

## Abstract

**Background:**

Existing standard non-molecular diagnostic methods such as viral culture and immunofluorescent (DFA) are time-consuming, labor intensive or limited sensitivity. Several multiplex molecular assays are costly. Therefore, there is a need for the development of a rapid and sensitive diagnosis of respiratory viral pathogens.

**Methods:**

A GeXP-based multiplex RT-PCR assay (GeXP assay) was developed to detect simultaneously sixteen different respiratory virus types/subtypes. Seventeen sets of chimeric primers were used to initiate the RT-PCR, and one pair of universal primers was used for the subsequent cycles of the RT-PCR. The specificity of the GeXP assay was examined with positive controls for each virus type/subtype. The sensitivity was evaluated by performing the assay on serial ten-fold dilutions of *in vitro*-transcribed RNA of all RNA viruses and the plasmids containing the Adv and HBoV target sequence. GeXP assay was further evaluated using 126 clinical specimens and compared with Luminex xTAG RVP Fast assay.

**Results:**

The GeXP assay achieved a sensitivity of 20–200 copies for a single virus and 1000 copies when all of the 16 pre-mixed viral targets were present. Analyses of 126 clinical specimens using the GeXP assay demonstrated that GeXP assay and the RVP Fast assay were in complete agreement for 109/126 (88.51%) of the specimens. GeXP assay was more sensitive than the RVP Fast assay for the detection of HRV and PIV3, and slightly less sensitive for the detection of HMPV, Adv, RSVB and HBoV. The whole process of the GeXP assay for the detection of 12 samples was completed within 2.5 hours.

**Conclusions:**

In conclusion, the GeXP assay is a rapid, cost-effective, sensitive, specific and high throughput method for the detection of respiratory virus infections.

## Background

Viral respiratory tract infections, which have a considerable morbidity and fatality rate, are common diseases that especially affect infants and the elderly [1]. Common respiratory viruses include influenza A virus, influenza B virus, parainfluenza virus, human rhinovirus, adenovirus and respiratory syncytial virus. New respiratory viruses important to public health, such as metapneumovirus, coronavirus (subtypes SARS-Cov and CoV HKU1) and human bocavirus [2,3], have emerged over the past decade. The clinical presentation of respiratory infections caused by different viral pathogens can be very similar, making etiological diagnosis difficult [4].

The traditional assays used to diagnose respiratory tract viruses are viral culture and immunofluorescent staining. Viral culture remains the gold standard for the diagnosis of respiratory viruses because of its broad spectrum and high specificity. However, viral culture is time-consuming and has a low sensitivity for the detection of some respiratory viruses that have fastidious growth requirements [5,6]. Immunofluorescent staining is fast, but the assay sensitivity and the availability of antisera can be limiting factors [5,6]. With the development of rapid molecular techniques, molecular assays, especially in a multiplex format, have been accepted as tests of choice for broad spectrum detection of respiratory viruses. Several multiplex assays are available commercially such as xTAG RVP from Luminex [7,8], Multicode-PLx RVP from EraGen Biosciences [9], and ResPlex II from Qiagen [10]. However, all of them are based on a liquid-phase bead-based array technology to implement the detection, which has increased the cost and the implementation time of the whole assays.

The GenomeLab Gene Expression Profiler genetic analysis system (GeXP) developed by Beckman Coulter (Brea, CA, USA) is a new multitarget, high-throughput detection platform that integrates reverse transcription-PCR (RT-PCR) and labeled amplified products in a multiplex PCR assay, followed by fluorescence capillary electrophoresis separation based on the size of the amplified products. This system has been used previously in identifying rapidly gene expression prostate cancer biomarker signatures in biological samples, rapid and sensitive detection of 68 unique varicella zoster virus gene transcripts [11] and detection of pandemic influenza A H1N1 virus [12] and nine serotypes of enteroviruses associated with hand, foot and mouth disease [13].

In this report, a novel RT-PCR assay using the GeXP (GeXP assay) for rapid, sensitive, multiplex detection of sixteen different respiratory virus types/subtypes: influenza A virus (FluA), influenza B virus (FluB), seasonal influenza A H1N1 virus (sH1N1), parainfluenza virus type 1 (PIV1), parainfluenza virus type 2 (PIV2), parainfluenza virus type 3 (PIV3), human rhinovirus (HRV), human metapneumovirus (HMPV), adenovirus (Adv), respiratory syncytial virus A (RSVA), respiratory syncytial virus B (RSVB), four coronavirus sybtypes (CoV HKU1, CoV NL63, CoV 229E, CoV OC43) and human bocavirus (HBoV) was described. The specificity and sensitivity of the GeXP assay were examined, and the clinical performance of the GeXP assay was evaluated by comparing the results obtained by the GeXP assay to those obtained by the Luminex xTAG RVP Fast kit (Luminex Corporation, Toronto, Canada) for 126 nasopharyngeal aspirates collected from hospitalized children.

## Methods

### Viruses and controls

Cell culture virus stocks from the NATtrol^TM^ Respiratory Validation Panel 2 (NATRVP-2) (ZeptoMetrix, New York, USA) were used as the positive controls for FluA H3, FluB, sH1N1, PIV1-3, HRV, Adv 3, RSVA, RSVB, HMPV, CoV 229E and CoV OC43. Clinical specimens that were genotyped and sequenced previously by the Biotech Center for Viral Disease Emergency, a part of the Chinese Center for Disease Control (CCDC), were used as positive controls for HBoV, CoV HKU1 and CoV NL63.

To evaluate the sensitivity of the assay, controls were prepared with dilutions from 10 to 10^5^ copies of vectors containing PCR products cloned from each virus individually. The PCR products were cloned into a pGEM-T vector, which was used to transform DH10B cells. The plasmid DNA was extracted with an E.Z.N.A. Plasmid Mini Kit I (Omega, GA, USA). The RNA copy number was calculated after measuring the concentration of the RNA transcribed *in vitro* using a T7 Large Scale RNA Production System (Promega, Wisconsin, USA). For the DNA viruses Adv and HBoV, the *in vitro* transcription was omitted.

### Clinical specimens

A total of 126 nasopharyngeal aspirates were collected from children under two years of age who were hospitalized at the Children’s Hospital of Beijing, China, during June, 2008 and March, 2010 with a diagnosis of pneumonitis or bronchopneumonia and a fever of 38°C or greater. A total volume of 0.5 ml of nasopharyngeal aspirate was collected in 3.5 ml of transport medium. This study was approved by the Beijing ethics committee. Sample collection was agreed by child's parents or grandparents with informed consent.

### Primer design

In total, 17 pairs of chimeric primers, one pair of internal control primers and one pair of universal primers were designed for the RT-PCR. The chimeric primers consisted of a gene-specific sequence fused at the 5’ end to the universal sequence. The specific sequences were selected by alignment of all sequences available for each virus from the National Center for Biotechnology Information (NCBI) GenBank Database. Primers for the human RNase P gene were used as an internal control for the RT-PCR of the clinical specimens. The forward universal primer was Cy5-labeled at the 5’ end of the sequence. The primers sequences, their target genes and the size of the resulting amplicons are listed in Table 1[14-16].

**Table 1:** Primer information

**Primer**	**Sequence 5’-3’**	**Gene**	**Size (bp)**
FluA F	AGGTGACACTATAGAATATTCTAACCGAGGTCGAAACG	M	270
FluA R	GTACGACTCACTATAGGGAACAAAGCGTCTACGCTGCAG		
FLuB F	AGGTGACACTATAGAATAAAAAGRAGATTCATCACAGAGC	M	166
FLuB R	GTACGACTCACTATAGGGATTCTGCTATTTCAAATGCTTCA		
sH1N1 F	AGGTGACACTATAGAATAGGTATGCTTTTGCAMTGARTAGAGG	HA	250
sH1N1 R	GTACGACTCACTATAGGGAAAGGGATATTCCTTARTCCTGTARCCAT		
PIV1 F	AGGTGACACTATAGAATATCTCATTATTACCYGGACCAA	HA	284
PIV1 R	GTACGACTCACTATAGGGATCCTGTTGTCGTTGATGTCATA		
PIV2 F	AGGTGACACTATAGAATATCTACACTGCATCAGCCAGC	HA	194
PIV2 R	GTACGACTCACTATAGGGACCCCTAAAAGAGATGAGCCC		
PIV3 F	AGGTGACACTATAGAATATTGTCAATTATGATGGYTCAATCT	HA	230
PIV3 R	GTACGACTCACTATAGGGAGACACCCAGTTGTGTTGCAG		
HRV F	AGGTGACACTATAGAATACCCCTGAATGYGGCTAACCT	5' UTR	144
HRV R	GTACGACTCACTATAGGGACGGACACCCAAAGTAGTYGGT		
HMPV F1	AGGTGACACTATAGAATACATGCCCACTATAAAAGGTCAG	L	208
HMPV R1	GTACGACTCACTATAGGGACACCCCAGTCTTTCTTGAAA		
HMPV F2	AGGTGACACTATAGAATAGAGCTAAYAGAGTGCTAAGTGATG	N	208
HMPV R2	GTACGACTCACTATAGGGAACTTTCTGCTTTGCTTCCTGT		
Adv F	AGGTGACACTATAGAATAGCCSCARTGGKCWTACATGCACATC	Hexon	338
Adv R	GTACGACTCACTATAGGGACAGCACSCCICGRATGTCAAA		
NL63 F	AGGTGACACTATAGAATATCCCAAATGTGATAGAGCTTTGC	Polym-erase	176
NL63 R	GTACGACTCACTATAGGGACTGTTAAAACTTGTGCCAACTC		
OC43 F	AGGTGACACTATAGAATAATTGCACCAGGAGTCCCA	N	200
OC43 R	GTACGACTCACTATAGGGATATCGGTGCCGTACTGGTCT		
229E F	AGGTGACACTATAGAATACTCGGAATCCTTCAAGTGACAGA	N	183
229E R	GTACGACTCACTATAGGGAACGAGAAGGCTTAGGAGTAC		
HKU1 F	AGGTGACACTATAGAATATATAGTRAAACCTGATATGGCT	N	220
HKU1 R	GTACGACTCACTATAGGGATACCAAAACACTGTTGAACAT		
RSVA F	AGGTGACACTATAGAATACATCCCCTCTATGCACAACC	F	158
RSVA R	GTACGACTCACTATAGGGACATGTTTCAGCTTGTGGGAA		
RSVB F	AGGTGACACTATAGAATAAAACGAAGATTTCTGGGCTTC	F	279
RSVB R	GTACGACTCACTATAGGGATGCGACAGCTCTGTTGATTT		
HBoV F	AGGTGACACTATAGAATAAAGAAAAGGGAGTCCAGAA	NP1	290
HBoV R	GTACGACTCACTATAGGGACTCTGTGTTGACTGAATACAG		
Rnasep F	AGGTGACACTATAGAATAGAGGCCTGGCTTTTGAACTT	RNase P	125
Rnasep R	GTACGACTCACTATAGGGAATCAAATTGAGGGCACTGGA		
Tag F	AGGTGACACTATAGAATA		
Tag R	GTACGACTCACTATAGGGA		

^a^ The primers HMPV F1 and HMPV R1 were reported previously [14]; the primers Adv F and Adv R were reported previously [15]; the primer HBoV R was reported previously [16]. The Universal Tag sequences are underlined.

### Extraction and purification of RNA/DNA

Total RNA/DNA was extracted from 200 μl of viral stocks or clinical samples using the QIAamp Viral RNA Mini Kit (Qiagen, Hilden, Germany) according to the manufacturer’s instructions. The extracts was eluted in 50 μl of DNase- and RNase-free water and stored at −80°C.

### GeXP assay and detection

The multiplex RT-PCR was performed with a One Step RT-PCR kit (Qiagen, Hilden, Germany) in a 25 μl volume, containing 5 μl of the 5× buffer, 1 μl of the dNTP mix, 1 μl of the enzyme mix, 1.25 pmol each of the forward chimeric primer mix and reverse chimeric primer mix, 12.5 pmol each of the forward universal primer mix and reverse universal primer mix, 2 μl of template RNA and 0.1 μl of ribonuclease inhibitor (Takara, Dalian, China) and DNase- and RNase-free water. The RT-PCR mixture was subjected to the following amplification conditions: 50°C for 30 min, 95°C for 15 min, followed by 10 cycles of 95°C for 30 s, 55°C for 30 s, 72°C for 30 s; 10 cycles of 95°C for 30 s, 65°C for 30 s, 72°C for 30 s; 20 cycles of 95°C for 30 s, 48°C for 30 s, 72°C for 30 s, and a final incubation of 72°C for 3 min. Two μl of each Cy5-labeled PCR product was separated via GeXP capillary electrophoresis, and the dye signal strength was measured by fluorescence spectrophotometry in arbitrary units (A.U.) of optical fluorescence. For all amplified products, the reaction was considered positive when the value of dye signal was over 2000 A.U.

### Specificity and sensitivity of the GeXP assay

The specificity of the GeXP assay for all viral targets was tested individually in a multiplex assay under the experimental conditions of the GeXP assay. The sensitivity of the GeXP assay for all viral targets was examined individually in a multiplex assay using serial ten-fold dilutions from 10 to 10^5^ copies of in vitro-transcribed RNA of all RNA viruses and the plasmids containing the Adv and HBoV target sequences. The sensitivity of the GeXP assay was also examined by using 16 pre-mixed quantitative viral targets.

### RVP fast assay and detection

The Luminex xTAG RVP Fast kit (Luminex Corporation, Toronto, Canada) enables users to detect simultaneously FluA, FluB, RSV, PIV1-4, AdV, HMPV, CoV 229E, NL63, OC43, and HKU1, enterovirus, HRV and HBoV. The RNA/DNA extracted from 126 clinical specimens was tested using the RVP Fast assay in a 96-well plate format according to the manufacturer’s instructions (Luminex Corporation, Toronto, Canada). The plate was then analyzed using the Bio-Plex^TM^ 200 system (Bio-Rad, CA, USA) according to the manufacturer’s instructions, and the median fluorescence intensity (MFI) was determined.

## Results

### Specificity and sensitivity of the GeXP assay

**Figure 1:**
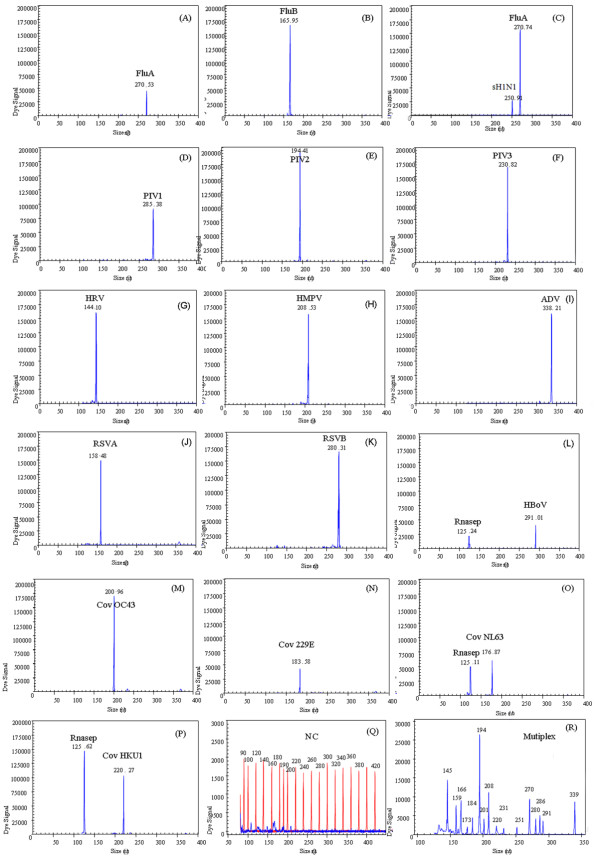
**Specificity and sensitivity analyses of the GeXP Assay.** The Y-axis indicates the dye signal in arbitrary units, and the X-axis indicates the actual PCR products size. Panels A-P show the results of amplification of FluA (H3N2), FluB, FluA (sH1N1), PIV1, PIV2, PIV3, HRV, HMPV, Adv, RSVA, RSVB, HBoV, CoV OC43, CoV 229E, CoV NL63 and CoV HKU1, respectively. Nuclease-free water was used as a negative control (NC). All of the 16 pre-mixed viral targets could be detected at the level of 1000 copies of each virus per reaction in the multiplex assay (R). The viral targets, from left to right, are HRV, RSVA, FluB, CoV NL63, CoV 229E, PIV2, CoV OC43, HMPV, CoV HKU1, PIV3, sH1N1, FluA, RSVB, PIV1, HBoV and Adv (R).

The specificity of the GeXP assay for all viral targets was tested individually in a multiplex assay. No mispriming was observed when all 17 pairs of the chimeric primers and one pair of the internal control primers were mixed together. The specific products could be obtained and separated clearly from the other viral targets on the GeXP system for all of the positive controls (Figure 1. A-P). For the viral targets that used clinical specimens as positive controls, including HBoV, CoV HKU1, and CoV NL63, a single PCR product was detected in addition to the internal control peak. The dye signal in the analysis was above 25000 A.U.

The sensitivity of the assay was evaluated individually for each virus in the multiplex assay using serial ten-fold dilutions of cloned PCR products. In the multiplex assay, the detectable level of HRV and PIV2 was 20 copies per reaction, while the limit of detection for the other 14 viruses types/subtypes was 200 copies per reaction. The clones with the same copies of each virus were then pre-mixed to evaluate the sensitivity when all of the 16 viral targets were present. The detection sensitivity of all of the pre-mixed viral targets was 1000 copies per reaction in the multiplex assay (Figure 1. R).

### Evaluation of the GeXP assay using respiratory specimens

All 126 specimens detected by the RVP Fast assay, the reference method, were retested by the GeXP assay. The results from both assays showed that HRV was found most frequently (68/126), followed by RSVB (46/126) and PIV3 (21/126) in the 126 specimens. A total of 23 negative specimens (18.25%) were detected by the GeXP assay and 24 (19.05%) negative specimens by the RVP Fast assay. One negative specimen detected by the RVP Fast assay was found to be positive for HRV by the GeXP assay. A total of 66 specimens with co-infections were detected by the RVP Fast assay, 64 of them were co-infections detected by the GeXP assay, and 4 additional co-infections were detected only by the GeXP assay and confirmed by sequencing as true positives.

The results from the GeXP assay and the RVP Fast assay were in complete agreement for 109/126 (86.51%) of the specimens. As shown in Table 2, there were 13 additional viruses (3 HRV, 3 RSVB, 2 HMPV, 1 Adv and 4 HBoV in 9 specimens) found only in the RVP Fast assay and 9 viruses (6 HRV and 3 PIV3 in 8 specimens) found only in the GeXP assay. All of the 9 additional viruses detected only by the GeXP assay were confirmed by sequencing as true positives. The results showed that the GeXP assay was more sensitive than the RVP Fast assay for the detection of HRV and PIV3, and less sensitive for the detection of HMPV, Adv, RSVB and HBoV. For RSVA, CoV, FluA, FluB, sH1N1, PIV1, and PIV2, both the sensitivity and specificity were 100%. The sensitivity, specificity, negative prediction value (NPV) and positive prediction value (PPV) of each virus, when compared to RVP Fast assay as a reference, were calculated for the GeXP assay in Table 2.

**Table 2:** Detection of 16 respiratory viruses in 126 specimens

**Virus**	**No. of specimens**				**Performance of the GeXP assay**			
	**GeXP** + ^**a**^**RVP** + ^**a**^	**GeXP** + **RVP -**	**GeXP- RVP +**	**GeXP-**^**a**^**RVP -**^**a**^	**Sensitivity %**	**Specificity %**	**PPV**^**a**^**%**	**NPV**^**a**^**%**
FluA	5	0	0	121	100	100	100	100
sH1N1^b^	3	0	0	123	100	100	100	100
FluB	1	0	0	125	100	100	100	100
PIV1	1	0	0	125	100	100	100	100
PIV2	1	0	0	125	100	100	100	100
PIV3	21	3^d^	0	102	100	97.14	87.50	100
HRV^c^	68	6	3	49	95.77	89.09	91.89	94.23
HMPV	8	0	2	116	80.00	100	100	98.31
Adv	9	0	1	116	90.00	100	100	99.15
CoV NL63	1	0	0	125	100	100	100	100
CoV OC43	12	0	0	114	100	100	100	100
CoV 229E	2	0	0	124	100	100	100	100
CoV HKU1	4	0	0	122	100	100	100	100
RSVA^b^	2	0	0	124	100	100	100	100
RSVB^b^	46	0	3	77	93.88	100	100	96.25
HBoV	15	0	4	107	78.95	100	100	96.40

^a^ The numbers of positive and negative specimens detected by the GeXP assay are indicated as GeXP + and GeXP -, respectively, and the numbers of positive and negative specimens detected by the RVP Fast assay are indicated as RVP + and RVP -, respectively. The sensitivity (True positives, TP), specificity (True negatives, TN), PPV (TP/TP + false positives), and NPV (TN/TN + false negatives) for each target are calculated using the RVP Fast assay as the reference for comparison.

^b^ The RVP Fast assay was able to subtype the FluA and RSV positive specimens.

^c^ The RVP Fast assay was not able to distinguish rhinovirus from enterovirus, so ‘HRV’ positive detected by the RVP Fast assay could be HRV or enterovirus. All of the specimens positive for HRV detected by the GeXP assay were confirmed by sequencing as true positives.

^d^ All of the additional PIV3 detected only by the GeXP assay were confirmed by sequencing as true positives.

## Discussion

A one-step, sensitive, specific assay using GeXP for the simultaneous detection of 16 respiratory virus types/subtypes (FluA, FluB, sH1N1**,**PIV1, PIV2, PIV3, HRV, RSVA, RSVB, HMPV, Adv, CoV OC43, CoV 229E, CoV NL63, CoV HKU1 and HBoV) was described in this study. The GeXP genetic analysis system is a new multitarget, high throughput detection platform, and its application in the differential detection of pandemic Influenza A H1N1 virus and seasonal Influenza A virus was reported recently by our laboratory [12]. To further reduce the assay time and avoid contamination, a one-step multitarget RT-PCR was performed in one tube rather than multiplex two-step RT-PCRs in separate tubes [12] would be preferred. To develop a one-step GeXP assay, several commercial one-step RT-PCR kits from different companies (Omega, Bio-Rad, Qiagen and Invitrogen) were tested in our preliminary experiments. The one-step RT-PCR kit from Qiagen revealed the best amplification to perform under our current protocol (data not shown).

The temperature switch PCR (TSP) [17] strategy was adopted to optimize the amplification parameters. The biphasic PCR parameters of the TSP allow a multiplex PCR to be performed under standardized PCR conditions, and therefore do not require optimization of each individual PCR assay. The optimal settings for three different denaturation temperatures and the amplification cycle conditions were determined in the current protocol. The chimeric primers consisted of a specific primer sequence fused to the universal primer sequence. The primers were designed to generate gene fragments with lengths between 140–340 bp. The amount of universal primers included in the RT-PCR was ten times that of the chimeric primers, so in the last 20 cycles of PCR, amplification was carried out predominantly by a single pair of universal primers, of which only the forward universal primer was labeled fluorescently. This should reduce the occurrence of preferential amplification in the reaction. Internal control primers were added to the reaction to ascertain whether the extraction and reverse-transcription steps of the assay were functioning correctly. Only 2 μl of the cy5-labeled PCR amplified fragments were separated by capillary electrophoresis based on size and detected by the GeXP system.

The specificity of the GeXP assay was examined using artificial specimens from the NATRVP-2 panel and clinical specimens confirmed previously to be positive. A specific peak of PCR product was obtained only for the expected viral target using the GeXP system. No cross-reactivity among the 16 respiratory virus types/subtypes was observed. The detection sensitivity for each virus was 20–200 copies per reaction when the assay was performed separately for each virus, and the sensitivity was 1000 copies per reaction when all of the 16 pre-mixed viral targets were present in the multiplex assay. The results indicate that the detection sensitivity of the GeXP assay for mixed virus samples was slightly lower than that for a single type of virus. The detection sensitivity for each virus in this study was similar to that of real-time PCR assays reported in recent years [18-20].

Analyses of 126 specimens using the GeXP assay and RVP Fast assay demonstrated that the GeXP assay had comparable sensitivity and specificity to the commercially available RVP Fast assay (Table 2). Discrepancies in detection results were found for HRV, PIV3, HMPV, Adv, RSV B and HBoV. All of the 9 specimens positive for PIV3 or HRV detected only by the GeXP assay were confirmed by independent PCR and sequencing to be true positives (data not shown), suggesting that the GeXP assay is more sensitive than the RVP Fast assay for the detection of HRV and PIV3. Because the HRV primers used in the RVP Fast assay were able to amplify both HRV and enterovirus, the three specimens positive for HRV detected only by the RVP Fast assay could actually be enteroviruses. All of the specimens positive for HRV detected by the GeXP assay were confirmed by sequencing as true HRV positives. Two of HMPV negative samples, 1 of Adv negative sample, 3 of RSVB negative samples and 4 of HBoV negative samples detected by the GeXP assay were positive by the RVP Fast assay. All of these negative samples had lower median florescence intensity (MFI) values (294–825) in the RVP Fast assay, suggesting that the GeXP assay has a slightly decrease sensitivity for the detection of HMPV, Adv, RSVB and HBoV. However, the difference was not significant (for HMPV, Adv, RSVB and HBoV, *p =* 0.5, 1, 0.25, 0.125, respectively, using McNemar’s test; Kappa = 0.880, 0.943, 0.949, 0.864, respectively). The detection of coronaviruses (CoV HKU1, CoV NL63, CoV 229E and CoV OC43) by both assays was completely consistent between assays. The overall detection rate of the GeXP assay for each virus was comparable to that of the RVP Fast assay, demonstrating the high sensitivity and specificity of the GeXP assay in the analysis of clinical samples. It should be noted that there are not sufficient positive samples for FluA, sH1N1, FluB, PIV1,PIV2, CoV NL63, CoV 229E, CoV HKU1 and RSVA to determine meaningful statistics in our study. Due to the limited specimens available at this time, only preliminary findings were reported in this study. The detection rate for infections involving two or more viruses was similar for the GeXP assay (68/126) and the RVP Fast assay (66/126). Most occurrences of multiplex infections involved two of three viruses, HRV, RSVB and PIV3 (data not shown).

Two distinct advantages of the GeXP assay are the short assay time and the low cost per test. Though the cost of GeXP equipment is approximately $100.000, the cost of the GeXP assay for simultaneous detection of 16 respiratory virus types/subtypes is approximately $8 per test (the RT-PCR kit and the consumables of detection), versus $120 per test using the RVP Fast kit or $8 per test for each virus using a commercial RT-PCR kit (DaAn, Gene, China). The whole reaction was completed in one tube in a one-step multiplex RT-PCR within 2.5 hours, followed by capillary electrophoresis separation (10 min/12 wells). In addition, two 96-well plates can be placed in parallel in a GeXP machine at the same time to further increase the throughput of the samples.

## Conclusions

In summary, this study has demonstrated that the GeXP assay is a rapid, cost-effective and high throughput method with high sensitivity and specificity for the detection of respiratory virus infection. Further validation of the GeXP assay with a larger number of clinical samples is necessary before this method can be used widely for routine laboratory testing in China.

## Competing interests

The authors declare that they have no competing interests. A Chinese patent (application number: 201110168721.X) has been filed for the combination of all of the primers specific to the 16 respiratory tract viruses listed in this study and the experimental parameters used for the RT-PCR assay. The authors Xue-jun Ma and Wen-bo Xu are inventors on the patent application. This technology is available for research only.

## Authors’ contributions

JL and NM performed the operation of GeXP experiment. CZ was responsible for providing the clinical samples and leading the evaluation of GeXP result using RVP method with JL, MY and MW. Dr. WX offered great help in the supplement of different clinical samples as well as shared previous experimental data. Dr. XM designed and coordinated the study, analyzed data and drafted the manuscript with JL. All authors read and approved the final version of this manuscript.

## Pre-publication history

The pre-publication history for this paper can be accessed here:

http://www.biomedcentral.com/1471-2334/12/189/prepub
